# Polymeric Micelle Systems for Oral Drug Delivery of Small Molecule Therapeutics

**DOI:** 10.3390/pharmaceutics18060744

**Published:** 2026-06-16

**Authors:** Eungyeop Lee, Jum Bum Kwon, Hyuk Jun Cho, Mi Ran Woo, Dong Wuk Kim, Jong Oh Kim, Duhyeong Hwang

**Affiliations:** 1 BK21 FOUR Community-Based Intelligent Novel Drug Discovery Education Unit, Vessel-Organ Interaction Research Center (VOICE, MRC), Research Institute of Pharmaceutical Sciences, College of Pharmacy, Kyungpook National University, Daegu 41566, Republic of Korea; sa03103@naver.com (E.L.); dkim17@knu.ac.kr (D.W.K.); 2 College of Pharmacy, Yeungnam University, Gyeongsan 38541, Republic of Korea; kgb0703@hanmail.net; 3 College of Pharmacy, Keimyung University, Daegu 42601, Republic of Korea; hjcho89@kmu.ac.kr; 4 Department of Pharmaceutical Engineering, Daegu Catholic University, Gyeongsan 38430, Republic of Korea; mrwoo95@cu.ac.kr

**Keywords:** oral bioavailability, small molecule drug, polymeric micelle, gastrointestinal stability, drug delivery

## Abstract

Oral administration remains the most convenient and favored route for systemic delivery of small-molecule drugs, primarily due to patient compliance and the absence of invasive procedures. Yet, poor aqueous solubility, chemical/enzymatic instability, and limited permeability in the gastrointestinal (GI) tract often result in low bioavailability (BA) of many therapeutic agents. Polymeric micelles formed from the self-assembly of amphiphilic block copolymers have gained considerable attention as a nanotechnology-driven solution to overcome these challenges. Their hydrophobic core–hydrophilic shell structure enables efficient encapsulation of poorly soluble small molecule drugs, providing protection from acidic or enzymatic degradation while potentially enhancing drug transport across the intestinal epithelium. This review examines the design principles, formulation strategies, and in vivo performance of polymeric micelles for oral delivery of small molecule drugs. We discuss strategies to improve micelle stability in the GI environment, including optimization of core hydrophobicity, kinetic stabilization, and corona engineering, and compare polymeric micelles with established alternatives such as self-micro emulsifying drug delivery system (SMEDDS) and amorphous solid dispersions (ASDs) across critical performance parameters. Despite decades of preclinical progress, no oral polymeric micelle formulation has reached regulatory approval, underscoring the persistent challenge of maintaining micellar structural integrity under the dynamic conditions of the GI environment. This review therefore examines not only the promise but also the structural vulnerabilities of oral micelles, proposing a stability-centered framework for interpreting micelle function under GI conditions. Finally, we discuss current translational challenges and suggest directions for future research toward clinical application of oral polymeric micelle systems.

## 1. Introduction

Oral drug delivery is the most widespread and patient-friendly method for administering pharmaceutics. Advantages such as non-invasiveness, self-administration, and reduced costs of clinical oversight make it highly desirable for a broad range of therapeutic applications. Nonetheless, oral route inefficiencies often reduce the effective dose reaching systemic circulation. Among currently approved small molecule drugs, approximately 40% of new chemical entities exhibit poor water solubility, and an estimated 70% of drug candidates in development face some degree of solubility-limited BA [1]. Moreover, small molecules that are chemically labile or metabolically vulnerable such as peptides and some complex small-molecule constructs are prone to significant degradation in the acidic gastric environment and are targets of intestinal and hepatic first-pass metabolism [2,3]. The recent approval of oral semaglutide (Rybelsus^®^) has demonstrated the clinical feasibility of oral peptide delivery, although significant pharmacokinetic and formulation challenges remain [4]. To surmount the hurdles of low solubility and inadequate GI stability, the pharmaceutical field has devoted substantial effort to the development of advanced nanocarrier systems, including polymeric nanoparticles, emulsions, solid lipid nanoparticles [5,6], liposomes, and polymeric micelles [7,8]. Among these, polymeric micelles have attracted considerable attention as a versatile nanocarrier platform [9,10]. polymeric micelles are formed by the self-assembly of amphiphilic block copolymers above their critical micelle concentration (CMC) in aqueous media; hydrophobic segments aggregate into a compact core, while hydrophilic segments extend outward to form a stabilizing corona [11,12,13]. The resulting core–shell nanostructure, typically 20–100 nm in diameter, provides a hydrophobic reservoir capable of encapsulating poorly soluble drug molecules. These physicochemical characteristics can be advantageous for oral delivery. The hydrophobic core protects labile drugs from acidic and enzymatic degradation in the GI lumen, the hydrophilic corona can facilitate mucus penetration and interaction with the intestinal epithelium, and the nanoscale dimensions may promote uptake through enterocyte-mediated endocytosis or lymphatic transport pathways [14]. Originally developed for intravenous cancer chemotherapy, polymeric micelles are now being actively investigated for oral administration as a means to enhance the BA of poorly soluble small-molecule therapeutics.

Despite the extensive preclinical literature on oral polymeric micelles, a critical question remains insufficiently addressed whether the micellar nanostructure is maintained during GI transit or rapidly dissociates into free polymer and drug upon encountering bile salts, pH gradients, and dilution [15,16]. This distinction is not trivial, as it determines whether polymeric micelles function as nanocarriers or as solubilizers [8,17]. Existing reviews have predominantly focused on polymer chemistry and drug-loading optimization, often treating in vivo stability as a secondary consideration [18,19]. However, accumulating evidence from Förster resonance energy transfer (FRET)-based integrity studies [17,20], bile salt competition assays [21], and pharmacokinetic correlations increasingly demonstrates that structural persistence—not merely solubilization capacity—is the principal determinant of whether a polymeric micelle confers a nanocarrier advantage. This review therefore adopts a stability-centered framework to critically reassess the oral polymeric micelle field.

Prior reviews primarily organized the field by polymer type or application area and treated micelles as solubilization platforms [13,14,15]. Our review frames the entire discussion around the stability–function relationship, introducing the comparison of a “solubilizer vs. nanocarrier” continuum that determines whether micelles provide dissolution enhancement or nanocarrier-mediated transport (transcytosis, lymphatic uptake). Additionally, we provide the systematic cross-platform comparison through the lens of stability parameters (CMC, kinetic stability, core crystallinity), and the direct benchmarking against SMEDDS and ASDs with decision-making criteria for formulation scientists. The literature reviewed in this article was identified through systematic searches of PubMed, Web of Science, Scopus, and Google Scholar using the following keyword combinations: “polymeric micelle” AND (“oral delivery” OR “oral administration”) AND “small molecule”; “block copolymer micelle” AND “bioavailability”; and polymer-specific terms (poloxamers, pluronics, poly(ethylene glycol)–*b*–poly(lactic acid) (PEG–*b*–PLA), PEG–*b*–poly(ε-caprolactone) (PEG–*b*–PCL), PEG–*b*–poly(lactic-co-glycolic acid) (PEG–*b*–PLGA)) combined with “oral.” The search covered publications from 2000 to 2026, with emphasis on studies published from 2010 onward. Studies were included if they reported oral administration of polymeric micelles with in vivo pharmacokinetic outcomes (area under the curve(AUC), Cmax, or bioavailability) or characterized micelle stability under simulated or actual GI conditions. Studies focused exclusively on macromolecular therapeutics (proteins, peptides, or nucleic acids), purely intravenous formulations without oral relevance, and conference abstracts lacking full experimental data were excluded.

In this review, we first examine the GI barriers that challenge micelle integrity, including bile salt displacement, dilution-induced dissociation, pH gradients, and mucus interactions (Section 2). We then evaluate how thermodynamic and kinetic stability parameters collectively determine whether a micellar system functions as a nanocarrier or as a solubilization platform, integrating recent mechanistic insights from in vitro–in vivo correlation studies (Section 3). The major polymer platforms for oral micellar delivery such as poloxamers, pluronic, PEG–*b*–PLA, PEG–*b*–PCL, PEG–*b*–PLGA are then surveyed with emphasis on how their core architecture influences structural persistence under physiological conditions (Section 4). To contextualize these findings, polymeric micelles are benchmarked against SMEDDS and ASD across critical formulation and performance parameters (Section 5). Regulatory and translational considerations are discussed in Section 6, followed by critical perspectives that highlight fundamental conceptual limitations of current approaches and propose directions for future research (Section 7). By bridging polymer science, GI physiology, and translational pharmacology, this review aims to provide a comprehensive and critical resource for the rational development of oral polymeric micelle systems.

## 2. GI Barriers to Oral Drug Delivery

**Figure 1:**
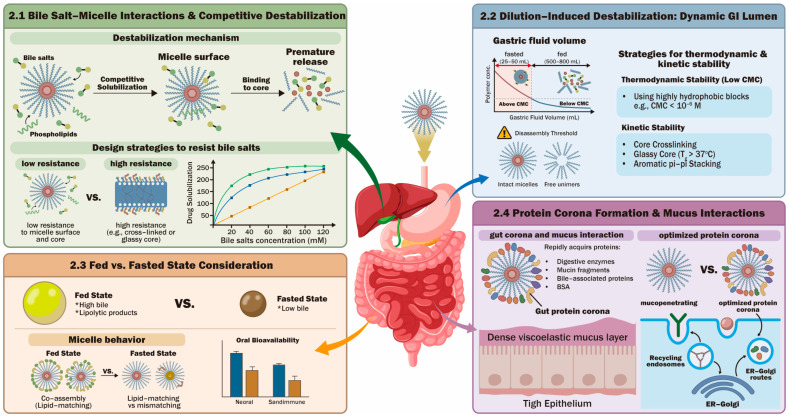
Dynamic GI barriers and design strategies for oral polymeric micelle drug delivery systems. The schematic illustrates the major destabilization mechanisms encountered during GI transit, including bile salt–micelle competitive interactions (Section 2.1), dilution-induced destabilization and prandial state effects (Section 2.2), and protein corona formation with mucus interactions (Section 2.3).

For orally administered polymeric micelles to deliver their cargo effectively, they must navigate a series of physiological barriers that are unique to the GI tract. Unlike the relatively controlled conditions encountered by intravenous formulations, oral micelles face dynamic and hostile environments including extreme pH gradients (pH 1.2–7.4), proteolytic and lipolytic enzymes, high ionic strength, endogenous bile salt surfactants, continuous dilution by GI secretions, a viscoelastic mucus barrier, and the tightly regulated intestinal epithelium [22,23]. Understanding these barriers at a mechanistic level is essential for rational micelle design (Figure 1).

### 2.1. Bile Salt–Micelle Interactions and Competitive Destabilization

Upon oral administration, polymeric micelles encounter bile salts in the duodenum and jejunum at concentrations up to 15 mM in the fed state [24]. As endogenous biosurfactants, bile salts can interact with amphiphilic assemblies and, under certain conditions, compete for hydrophobic drug incorporation, thereby altering the structure of the micellar core through mixed micelle formation and component redistribution, potentially leading to structural destabilization and premature drug release into the lumen [21,24].

Clulow et al. demonstrated that interactions between exogenous non-ionic surfactants and bile salt–phospholipid mixed micelles can produce synergistic, antagonistic, or additive effects on drug solubilization depending on surfactant structure [21]. In their study, relatively hydrophobic polymers such as Pluronic L81 and L121 enhanced poorly soluble fenofibrate solubilization by expanding the hydrophobic domain of the mixed micelles, whereas more hydrophilic surfactants polymers including Kolliphor EL, vitamin E D-α-tocopheryl polyethylene glycol 1000 succinate (TPGS), and Pluronic F127 reduced overall solubilization capacity despite increasing micelle size; highly hydrophilic polymers such as Pluronic F68 exhibited minimal interaction, resulting in largely additive behavior. Small-angle X-ray scattering (SAXS) analysis confirmed that both hydrophilic and hydrophobic surfactants induced structural reorganization and swelling of bile salt–phospholipid mixed micelles, yet only hydrophobic surfactants enhanced solubilization. These findings indicate that micelle size alone is not predictive of solubilization efficiency; rather, the internal hydrophobic character of the micellar core is the dominant determinant.

While SAXS provides structural insight into micelle reorganization, it does not directly capture the fate of polymeric micelles during GI transit. Direct evidence for the destabilizing impact of GI components on polymeric micelles has been provided by Sun et al., who employed FRET probes incorporated into mPEG–*b*–PCL micelles to monitor structural integrity in simulated gastric fluid, simulated intestinal fluid, and individual GI components [25]. Their results revealed that micelle destabilization arises from two distinct mechanisms: a “solubilization effect,” in which bile salts bind to the hydrophobic PCL core in a concentration-dependent manner, and a “hydration effect,” in which mucins penetrate through the PEG corona, particularly in micelles with low surface PEG density. Structural parameters including PCL block length, surface PEG density, particle size, and aggregation number were identified as key determinants of micelle integrity, with each factor exerting distinct effects on the two destabilization pathways. Longer PCL blocks and larger aggregation numbers enhanced resistance to bile salt-induced solubilization, while high surface PEG density and small particle size (<50 nm) mitigated mucin-induced integrity loss. Notably, the preparation method also proved critical: micelles produced by emulsification exhibited higher surface PEG density and more ordered PCL core arrangements than those prepared by nanoprecipitation, resulting in superior structural integrity. However, the study also revealed an inherent trade-off—high surface PEG density preserves structural integrity but impedes cellular uptake, creating an “easy uptake, hard transcytosis” dilemma that must be balanced in micelle design. Ultimately, cyclosporin A-loaded micelles with optimized structural integrity demonstrated enhanced oral BA and reduced hepatorenal toxicity compared to the commercially available Neoral^®^ formulation in vivo, establishing a direct link between micelle integrity and therapeutic performance.

These observations underscore the fact that the fate of orally administered polymeric micelles is determined not solely by their initial physicochemical properties but by the dynamic interplay between micellar architecture and the GI environment. Core properties such as hydrophobicity, crystallinity, and glass transition temperature govern resistance to bile salt-mediated solubilization, while corona characteristics including PEG density and chain length modulate interactions with endogenous bile salt–phospholipid structures [21,25]. Addressing this challenge requires deliberate optimization of core hydrophobicity, incorporation of stabilizing strategies such as core cross-linking, and corona engineering to minimize bile salt penetration—design principles that are discussed in detail in Section 3.

### 2.2. Dilution and Prandial State Effects on Micelle Stability

The GI lumen presents a dynamic dilution environment that challenges the structural stability of polymeric micelles. Following oral administration, formulations are progressively diluted by gastric and intestinal secretions, which can reduce the effective polymer concentration, often approaching or falling below the CMC, thereby promoting micelle destabilization or disassembly. Magnetic resonance imaging-based quantification in healthy human volunteers has demonstrated that resting gastric fluid volume in the fasted state is approximately 35 mL, with small intestinal fluid distributed in discrete pockets totaling only ~43 mL [26]; following a standard 240 mL water dose, gastric volume rises to ~242 mL and empties with a half-life of approximately 13 min, creating a rapidly changing dilution environment. This effect is particularly pronounced under fed-state conditions, where gastric fluid volumes can increase substantially beyond these baseline values, leading to significant dilution of the formulation [24].

Importantly, micellar systems in the GI tract operate under inherently non-equilibrium conditions. Continuous dilution, fluid turnover, and mixing are expected to hinder the establishment of a stable thermodynamic equilibrium between assembled micelles and free unimers. As a result, even systems initially formed above the CMC may undergo progressive structural rearrangement or disassembly during transit through the stomach and small intestine [26]. The susceptibility to dilution-induced destabilization is highly polymer-dependent: Polat et al. demonstrated that chitosan-based micelles maintained their particle size even after 1000-fold dilution, whereas synthetic Pluronic P123 micelles exhibited significant structural changes under comparable conditions, although the natural polymer micelles showed greater vulnerability to protein-mediated destabilization [27]. In addition to dilution, this destabilization is further exacerbated by simultaneous exposure to bile salts and digestive components, which can accelerate micelle disruption through competitive solubilization and mixed micelle formation [27].

These observations highlight that dilution-induced destabilization represents a fundamental barrier to oral polymeric micelle delivery. Under GI conditions, micelle integrity cannot be assumed based solely on initial formulation parameters, and systematic assessment using biorelevant media such as FaSSIF (Fasted State Simulated Intestinal Fluid) and FeSSIF (Fed State Simulated Intestinal Fluid) is essential to predict micellar behavior under physiologically representative dilution and surfactant conditions [11]. The ability of micelles to persist during the 3–5 h intestinal transit window becomes a critical determinant of their functional performance. The magnitude and consequences of this dilution effect are strongly modulated by the prandial state, as discussed below.

The prandial state influences the GI environment and the performance of oral polymeric micelles. In the fed state, increased bile salt secretion (up to 15 mM), elevated lipid content from dietary intake, and altered gastric pH and motility patterns create a significantly different milieu compared to fasting conditions [24]. Fed-state intestinal fluid contains higher concentrations of bile salt–phospholipid mixed micelles that can interact with polymeric micelles through competitive solubilization and co-assembly mechanisms [21,24].

Direct in vivo evidence for bile acid-dependent drug release from polymeric micelles was provided by Hasselt et al., who demonstrated that the oral absorption of vitamin K encapsulated in mPEG_5000_-b-p(HPMAm-lac_2_) thermosensitive micelles was virtually abolished in bile duct-ligated rats (AUC 1.64 ng/mL·h) compared with sham-operated controls (AUC 4543 ng/mL·h, *p* < 0.01), and was fully restored upon duodenal co-administration of exogenous bile acids [28]. Dynamic light scattering analysis further revealed a dose-dependent increase in micellar size in the presence of bile acids, consistent with gradual mixed micelle formation and drug transfer from the polymeric carrier to endogenous bile salt micelles [28]. These findings indicate that bile salts in the fed state not only destabilize polymeric micelles but also serve as essential mediators of drug extraction and transfer to absorptive mixed micelles, highlighting a dual role that complicates prandial state effects on oral polymeric micelle performance.

Although the fed state may paradoxically improve BA for poorly water-soluble drugs by providing additional solubilization capacity through endogenous mixed micelles, it also introduces substantial variability that complicates dosing regimen design. For example, the oral BA of cyclosporine A from Neoral^®^ microemulsion formulation shows reduced food-effect variability compared to earlier formulations, illustrating how formulation design can mitigate prandial state dependencies [29]. Similarly, the solubility of fenofibrate with Pluronic F127 increased approximately 1.8-fold due to enhanced bile salt-mediated solubilization, a pattern that polymeric micelle formulations must account for during development [21].

Thus, the fed state introduces competing effects on oral polymeric micelle performance: while solubilization may be enhanced, micelle stability may be simultaneously compromised due to complex interactions with bile salts and digestion products. As a result, oral polymeric micelle systems must be designed to maintain structural integrity under both fasted and fed conditions to ensure consistent and predictable pharmacokinetic behavior.

### 2.3. Protein Corona Formation and Mucus Interactions

Protein corona formation, considered a phenomenon exclusive to systemically circulating materials, has been increasingly recognized as occurring in orally administered drug delivery systems as well [30,31,32]. When polymeric micelles enter the GI tract, they rapidly acquire a layer of adsorbed proteins and biomolecules termed the “gut corona” or “biocorona” that fundamentally alters their surface properties and biological identity [33]. Kihara et al. investigated gut corona formation on model SiO_2_ nanoparticles exposed to simulated and extracted bile fluids, revealing that bile salt and phospholipid mixed micelles adsorb onto nanoparticle surfaces in a distinctive raspberry-like structure with the resulting corona proteome being compositionally distinct from the original bile fluid and enriched in enzymes and structural proteins [33]. This gut corona may mask targeting ligands, alter surface charge, modify mucus penetration behavior, and influence cellular uptake mechanisms at the intestinal epithelium [30,31]. However, emerging evidence suggests that the GI protein corona is not merely an obstacle but may also confer functional advantages.

Disease-specific intestinal protein coronas have been shown to selectively enrich transepithelial transport-associated proteins such as immunoglobulin receptors and transferrin, thereby enhancing transcytosis and oral absorption of nanoparticles through receptor-mediated pathways [32]. Wu et al. demonstrated that nanoparticles incubated in pathological intestinal fluid acquired a protein corona enriched in proteins associated with vesicular trafficking and secretory pathways [32]. This disease-specific corona altered intracellular trafficking pathways, promoting transport through recycling endosome and ER–Golgi-mediated secretory routes, which enhanced transcytosis across the intestinal epithelium and was associated with increased oral absorption compared with nanoparticles bearing a healthy intestinal corona [32]. Importantly, the formation and composition of the protein corona are not solely dictated by the biological environment but are also strongly influenced by the physicochemical properties of the micelle itself. Polymer composition, core hydrophobicity, and surface chemistry may affect the affinity for specific proteins and biomolecules, thereby shaping the resulting corona profile. These material-dependent corona characteristics further influence mucus interactions, as surface hydration, PEG density, and charge collectively regulate the balance between mucoadhesion and mucopenetration. Furthermore, pre-coating nanoparticles with specific corona proteins (e.g., bovine serum albumin) can improve both mucus penetration and epithelial uptake [30,31], suggesting that deliberate corona engineering represents a promising yet underexplored strategy for oral polymeric micelle design.

While the protein corona reshapes the biological identity of polymeric micelles, these modified nanocarriers must still traverse the viscoelastic mucus barrier—a rate-limiting step that fundamentally governs epithelial access and oral bioavailability. Subramanian et al. reviewed strategies for engineering mucus-interactive nanocarriers, highlighting that the balance between mucoadhesion (prolonged residence) and mucopenetration (rapid transit to epithelium) depends on PEG density, molecular weight, and surface charge [34]. Dense PEG coronas (≥5 % surface PEG content, brush conformation) minimize mucin binding and facilitate rapid mucopenetration, whereas lower PEG densities allow mucoadhesive interactions that extend residence time but may trap particles in the mucus layer. Xu et al. further demonstrated that PEG surface density is a key parameter controlling mucus penetration efficiency, with an optimal PEG density range above which nanoparticles transition from mucoadhesive to mucopenetrating behavior [35].

These findings point to an emerging shift in perspective on oral nanoparticle design. Earlier studies focused predominantly on mucopenetration as the primary strategy for enhancing epithelial access, assuming that efficient mucus transit alone would translate into improved absorption [34,35]. However, recent investigations reveal that the protein corona acquired during GI transit actively modulates cellular uptake pathways, organ-specific biodistribution, and transcytotic efficiency [36]. Integrating these findings, the next generation of oral polymeric micelle systems should pursue a dual-optimization strategy that combines mucopenetration engineering with deliberate protein corona modulation, leveraging disease-specific or engineered corona compositions to enhance transepithelial transport while maintaining surface functionality during mucus permeation [32,37].

## 3. Oral Micelle Stability and Translational Implications

As established in Section 2, the GI environment poses formidable challenges to micellar structural integrity. Without structural persistence during exposure to bile salts, pH gradients, dilution, and enzymatic degradation, polymeric micelles function merely as transient solubilizers rather than true nanocarriers [15,16]. Understanding the thermodynamic and kinetic parameters that govern micelle stability is therefore essential for rational formulation design.

### 3.1. Thermodynamic and Kinetic Stability Under GI Conditions

The self-assembly of amphiphilic block copolymers into polymeric micelles is governed by the minimization of Gibbs free energy (ΔG = ΔH − TΔS) in aqueous environments. The thermodynamic driving force for micellization, commonly expressed as the standard free energy of micelle formation (ΔG°mic), typically ranges from −20 to −40 kJ/mol for block copolymer systems and is primarily determined by the length and hydrophobicity of the core-forming block [38]. Increasing core hydrophobicity generally results in more negative ΔG°mic values and correspondingly lower CMC values. In the context of oral delivery, the CMC serves as a key translational stability parameter. Polymers with more hydrophobic or densely packed core-forming segments can achieve lower CMC values, often in the range of 10^−6^–10^−7^ M [9,14], thereby reducing the likelihood of complete disassembly under dilution conditions encountered in the GI tract. Importantly, CMC is primarily governed by the length and hydrophobicity of the core-forming block rather than by the polymer class per se; within any given polymer family, increasing the hydrophobic block molecular weight substantially lowers the CMC [9,11]. Because no systematic study has compared CMC values across different polymer types (e.g., PLA, PCL, PLGA cores) under identical block-length and molecular-weight conditions, cross-platform CMC rankings should be interpreted with caution.

However, thermodynamic stability alone is insufficient to ensure structural persistence under the non-equilibrium conditions described in Section 2.2. Kinetic stability, which defined by the rate of unimer exchange and micelle disassembly, plays a critical role in determining whether micelles remain intact during intestinal transit. Micelles with slow chain-exchange kinetics can persist for extended periods even when local concentrations fall below the CMC, provided that the activation barrier for dissociation is sufficiently high. Several structural features contribute to enhanced kinetic stability. Core crystallinity, high glass-transition temperatures (Tg), and strong intermolecular interactions such as hydrogen bonding or π–π stacking can significantly retard chain exchange [39,40].

**Figure 2:**
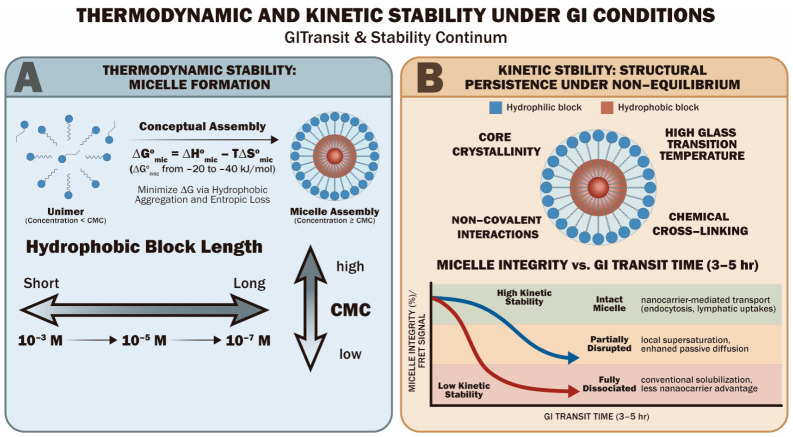
Thermodynamic and kinetic stability parameters governing oral polymeric micelle integrity. The relationship between core-block hydrophobicity and CMC (**left**, **A**) illustrates how increasing hydrophobic block length enhances thermodynamic stability, while kinetic stabilization strategies including core crystallinity, cross-linking, and glassy core formation (**right**, **B**) determine structural persistence during GI transit.

Collectively, these findings indicate that effective oral polymeric micelle design requires the simultaneous optimization of thermodynamic stability (low CMC) and kinetic stability (slow unimer exchange), rather than reliance on either parameter alone (Figure 2).

### 3.2. Formulation Strategies for Oral Polymeric Micelles

Several conventional methods are employed for the preparation of polymeric micelles [41], including direct dissolution, thin-film hydration, dialysis, emulsification–solvent evaporation, and spray-drying. The choice of preparation method strongly influences key formulation attributes such as particle size distribution, drug loading capacity, encapsulation efficiency, and the ability of the micellar system to reconstitute after processing. For oral drug delivery systems in particular, the preparation method must produce micelles that not only achieve adequate drug loading but also maintain colloidal stability upon dilution in GI fluids. Consequently, the selection of fabrication techniques has direct implications for the physicochemical characteristics that ultimately influence in vivo performance [42].

Among these approaches, direct dissolution represents the simplest method. In this strategy, the amphiphilic block copolymer is dissolved directly in aqueous media at concentrations above its CMC, allowing spontaneous self-assembly of micelles. Although operationally straightforward, this method often results in limited drug loading when highly hydrophobic compounds are used, primarily because the drug and polymer may not mix efficiently in the aqueous environment [42]. Thin-film hydration provides a practical solution to this limitation. In this method, the drug and polymer are co-dissolved in a volatile organic solvent that is subsequently evaporated to form a thin film, which is then rehydrated with aqueous media to induce micelle formation. The improved drug–polymer contact achieved during solvent evaporation generally results in higher encapsulation efficiencies, although reproducibility depends on careful control of film thickness and hydration conditions [42].

The dialysis method offers an alternative approach that enables gradual solvent exchange and controlled self-assembly. In this technique, the drug–polymer solution prepared in a water-miscible organic solvent is placed inside a dialysis membrane and dialyzed against an aqueous phase. As the organic solvent diffuses out and water diffuses in, micelles form progressively, often yielding systems with narrow particle size distributions. Despite these advantages, dialysis is relatively time-consuming and presents practical challenges for industrial-scale production [42]. Another commonly employed approach is emulsification–solvent evaporation, in which the drug and polymer are dissolved in a water-immiscible organic solvent and emulsified into an aqueous phase using sonication or high-shear homogenization. Subsequent removal of the solvent under reduced pressure promotes micelle formation. This method provides good control over particle size but requires careful removal of residual solvent to meet pharmaceutical specifications [42].

For oral administration, the conversion of liquid micellar dispersions into solid dosage forms is often necessary to improve patient compliance and ensure long-term storage stability. Spray-drying has proven particularly effective for this purpose [43,44], producing dry powders that can be readily filled into capsules or compressed into tablets while preserving the ability of the system to reconstitute into micelles upon hydration. This approach has also been extended to polymeric nano-aggregates, where spray-dried formulations of poorly soluble drugs demonstrated enhanced aqueous solubility and improved oral BA compared with unprocessed drug powder [45]. For example, spray-dried indomethacin-loaded polymeric micelles with particle sizes of approximately 7 µm reconstituted into micelles of roughly 130 nm while maintaining encapsulation efficiencies exceeding 80%, demonstrating that the self-assembled nanostructure can be retained through solid-state processing [43]. Similarly, lyophilization represents another strategy for stabilizing micellar dispersions, although the use of cryoprotectants such as trehalose is typically required to prevent irreversible aggregation during the freeze-drying process [46]. Beyond powders and lyophilizates, alternative solid dosage platforms such as oral disintegrating films have also been explored for rapid-dissolving delivery of poorly soluble drugs, offering additional options for patient-friendly oral formulations [47].

In addition to the preparation method, a critical formulation decision is the selection of drug–polymer pairs that maximize loading capacity and minimize premature drug release. Drug loading efficiency is governed by the polymer-to-drug ratio, the physicochemical properties of the drug (log P, pKa, and molecular size) [48], and the compatibility between the drug and the core-forming block. Drug–polymer compatibility can be predicted using the Flory–Huggins interaction parameter (χ), where lower χ values generally correspond to improved miscibility and higher equilibrium drug loading [49]. In practice, systematic matching of drug and polymer solubility parameters has been shown to substantially enhance formulation performance. For example, ellipticine loading in PEG–*b*–PCL micelles increased substantially when the solubility parameters of the drug and the core-forming block were closely matched, whereas formulations using PEG–*b*–PDLLA with a less compatible core exhibited rapid drug precipitation within 24 h [50].

Recent advances in manufacturing technologies have introduced microfluidic approaches as a next-generation alternative for micelle fabrication. Microfluidic mixing enables precise control over solvent exchange and nucleation kinetics during micelle formation, producing particles with narrower size distributions and improved batch-to-batch reproducibility compared to conventional bulk preparation methods [42]. These approaches may help address one of the key translational challenges for polymeric micelles scalable and reproducible manufacturing while maintaining the structural attributes required for stable oral drug delivery. Rather than relying solely on empirical formulation screening, rational design based on physicochemical matching and controlled fabrication strategies is essential for achieving both high drug loading and structural robustness under GI conditions [51,52].

### 3.3. In Vivo Performance and Mechanistic Considerations

Numerous preclinical studies have reported enhanced oral BA of poorly soluble drugs such as paclitaxel, cyclosporine A, and curcumin when formulated in polymeric micelles or in lipid formulations [14,53]. However, the mechanistic basis for these improvements varies considerably across formulations and is often poorly characterized. This section examines the pathways through which orally administered polymeric micelles enhance drug absorption, focusing on epithelial transport mechanisms, efflux transporter modulation, and the relationship between micelle integrity and pharmacokinetic performance.

**Figure 3:**
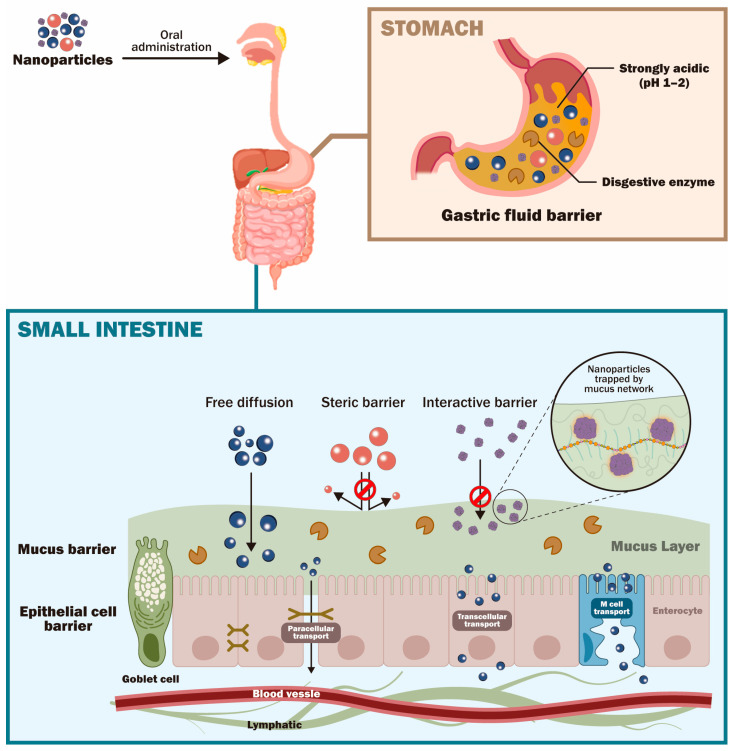
Graphical abstract illustrating the key challenges and design considerations for oral polymeric micelle drug delivery systems.

Structurally intact polymeric micelles may access multiple transport pathways to cross the intestinal epithelium, and the dominant pathway depends on the interplay between micelle properties and the local epithelial environment (Figure 3). Size influences the predominant uptake pathway: nanoparticles smaller than approximately 200 nm are generally internalized via clathrin-mediated endocytosis, whereas larger particles increasingly engage caveolae-dependent mechanisms, as demonstrated in non-phagocytic cell models [54]. However, the applicability of these size thresholds to intestinal enterocytes remains incompletely characterized, as caveolin-1 expression on the apical enterocyte membrane is debated and may vary along the intestinal tract [55,56]. Caveolae-mediated uptake offers the advantage of bypassing lysosomal degradation and thus preserving drug integrity during intracellular trafficking. Beyond enterocyte uptake, microfold (M) cells overlying Peyer’s patches provide a transcytotic shortcut that delivers intact nanoparticles to underlying lymphoid tissue, enabling lymphatic absorption and hepatic first-pass avoidance, which is a pathway especially relevant for lipophilic drugs with extensive hepatic metabolism [56]. Dense PEG coronas minimize non-specific mucin binding and favor mucopenetration to the epithelial surface [34,35]. In contrast, cationic polymers such as chitosan can transiently open tight junctions by redistributing claudin and occludin proteins, thereby enhancing paracellular transport of both the polymer and its encapsulated cargo [56]. Importantly, the relative contribution of each pathway varies along the intestinal tract, with Peyer’s patch-rich ileal regions favoring M-cell transcytosis and villous enterocytes dominating jejunal absorption [55,56].

In addition to epithelial transport mechanisms, modulation of efflux transporters represents another major contributor to enhanced oral bioavailability. P-glycoprotein (P-gp, ABCB1), located on the apical membrane of enterocytes, actively exports many hydrophobic drug molecules back into the intestinal lumen, thereby limiting oral absorption [57]. Certain amphiphilic polymers used in micelle formulations can inhibit this transporter. Pluronic block copolymers, for example, inhibit P-gp by inserting unimers into the lipid bilayer at concentrations below the CMC. This insertion increases membrane microviscosity and disrupts mitochondrial ATP production through inhibition of complexes I and IV, ultimately suppressing P-gp ATPase activity [57]. Among the poloxamer series, Pluronic P85 has shown particularly strong inhibitory effects, enhancing rhodamine-123 accumulation in Caco-2 monolayers [57]. TPGS acts through a complementary pathway involving inhibition of substrate-stimulated P-gp ATPase activity together with mitochondrial complex II inhibition [58]. In addition, chitosan-based micellar systems may exert dual effects by transiently modulating tight junction permeability while simultaneously reducing P-gp-mediated efflux, thereby facilitating both paracellular and transcellular drug transport [56].

Direct experimental evidence linking micelle integrity to in vivo pharmacokinetic performance has been provided by FRET-based studies. Building on earlier FRET-based tracking of micelle integrity in parenteral settings [59,60,61], Sun et al. extended this approach to oral delivery, conducting a mechanistically detailed study using mPEG–b–PCL micelles with varying hydrophobic block lengths to deliver cyclosporine A [25]. Their results showed that micelles with longer PCL blocks maintained significantly higher FRET efficiency during intestinal transit and yielded improved pharmacokinetic parameters (C_max_ = 757 ± 66 ng/mL; AUC_0–t_ = 21,938 ± 2184 ng·h/mL) [25]. These experiments identified two principal mechanisms of micelle destabilization during GI transit: the “hydration effect” of mucins, which weakens core packing through water penetration, and the “solubilization effect” of bile salts, which competitively extract hydrophobic polymer chains from the micellar core [25]. These findings provide mechanistic support for the observation that micelles with crystalline or high-Tg cores exhibit greater structural persistence in the GI environment.

The functional outcome of these processes can be conceptualized as a continuum linking micelle stability to drug absorption. Structurally persistent micelles that retain their core–shell architecture until reaching the epithelial surface may access nanoparticle-mediated transport pathways such as endocytosis or lymphatic uptake [55,56]. Partially disrupted micelles undergoing gradual unimer exchange may instead generate locally supersaturated drug concentrations near the epithelial interface, thereby enhancing passive transcellular diffusion beyond conventional dissolution limits [62]. Fully dissociated micelles, in contrast, release their cargo into the intestinal lumen and behave similarly to conventional solubilization systems, providing less nanocarrier-specific transport advantage [63].

Several quantitative examples illustrate the magnitude of BA enhancement achievable across this stability–function continuum. In a human pharmacokinetic study, Schiborr et al. reported that a polysorbate-based liquid micellar curcumin formulation (NovaSOL^®^) achieved a 185-fold increase in oral BA compared with native curcumin powder [64]. In preclinical studies, Pluronic P123/F127 mixed micelles improved the oral BA of lacidipine by approximately 6.85-fold relative to drug suspension [65], an enhancement partially attributed to P-gp inhibition by the poloxamer carrier [66]. It should be noted that in most of these studies, the contribution of intact nanocarrier transport versus enhanced solubilization was not mechanistically deconvoluted. Supersaturated polymeric micelles based on Soluplus^®^ have also been reported to increase the oral BA of cyclosporine A to 134.6% relative to the commercial Neoral^®^ formulation, demonstrating that optimized micellar systems can approach or even exceed the performance of established lipid-based delivery technologies [67].

## 4. Polymers for Oral Polymeric Micelle Formulations

The four major polymer platforms discussed in this section are summarized in Table 1. Although poly(amino acid)-based systems (e.g., PEG–*b*–poly(aspartate), elastin-like polypeptides) offer attractive properties including enzymatic degradation into natural amino acids and tunable secondary structures, their oral pharmacokinetic data remain absent; they are therefore not discussed further here.

### 4.1. Pluronics (Poloxamers) Systems

Poloxamers (Pluronics) are amphiphilic PEO–*b*–PPO–*b*–PEO triblock copolymers that self-assemble in aqueous environments to form micelles with a hydrophobic polypropylene oxide (PPO) core and a hydrophilic polyethylene oxide (PEO) corona. In the context of oral drug delivery, their utility extends beyond simple solubilization, as certain poloxamers are known to modulate membrane fluidity and inhibit intestinal P-glycoprotein (P-gp), thereby reducing efflux-mediated drug loss [68,69,70]. Pluronic F127 has additionally been employed as a stabilizer in nanocrystal formulations for oral mucosal delivery, further illustrating the versatility of poloxamer-based systems across diverse drug delivery applications [71].

For example, genistein-loaded Pluronic F127 micelles prepared by a solid dispersion method exhibited a mean particle size of approximately 28 nm with a drug loading efficiency of 97.4% [70]. Compared with a genistein suspension, the micellar formulation resulted in significantly enhanced oral BA and elevated AUC values in rat models. The enhanced systemic exposure was attributed to increased apparent solubility within the PPO core, sustained release behavior that reduced luminal precipitation, and partial inhibition of efflux transporters that facilitated intestinal absorption [70]. Because genistein undergoes rapid metabolism and exhibits poor aqueous solubility, the micellar system provided both protective encapsulation and solubilization enhancement.

**Figure 4:**
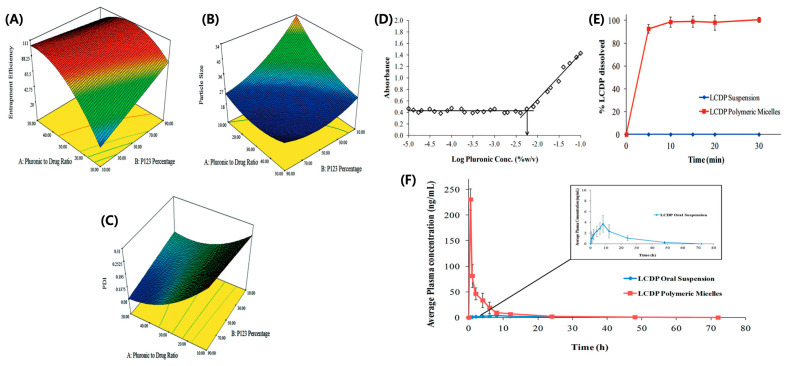
Formulation optimization and in vivo pharmacokinetic evaluation of lacidipine-loaded Pluronic P123/F127 mixed polymeric micelles. (**A**–**C**) Response surface plots from central composite design showing the effects of Pluronic-to-drug ratio and P123 percentage on encapsulation efficiency, particle size, and polydispersity index. (**D**) Critical micelle concentration determination of the optimized P123/F127 mixed system. (**E**) In vitro dissolution profiles comparing lacidipine polymeric micelles with lacidipine suspension, demonstrating rapid and complete release from the micellar formulation. (**F**) Mean plasma concentration–time curves following oral administration in rabbits, showing a 6.86-fold increase in oral BA for the micellar formulation relative to drug suspension. Reproduced with permission from Fares et al., Drug Delivery 2018 [65].

Lacidipine, a calcium channel blocker with extremely poor aqueous solubility, was formulated in Pluronic P123/F127 mixed polymeric micelles optimized through central composite design (Figure 4) [65]. The optimized formulation exhibited a particle size of approximately 21 nm, near-complete encapsulation efficiency (99.2%), and rapid dissolution exceeding 96% within 10 min. Following oral administration in rabbits, the micellar formulation achieved a 6.86-fold increase in oral BA compared with lacidipine suspension. The enhancement was attributed to the synergistic effects of P123-mediated P-gp inhibition and F127-mediated solubilization within the mixed micellar core.

**Figure 5:**
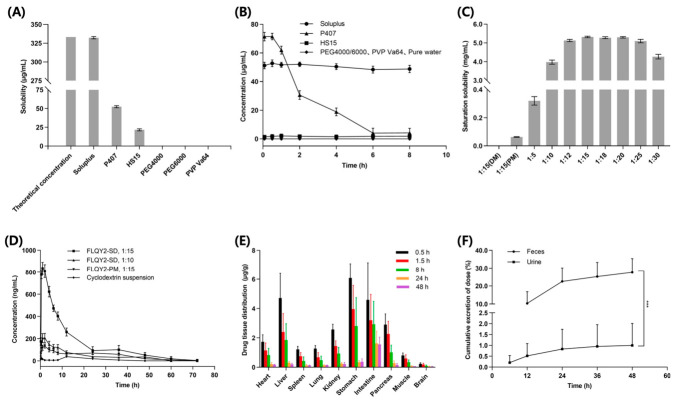
Characterization of FLQY2-loaded Soluplus^®^ self-micelle solid dispersion. (**A**) Solubility of FLQY2 in various polymer solutions, showing superior solubilization by Soluplus^®^ (~332 µg/mL). (**B**) Concentration–time profiles of FLQY2 released from different polymer solutions, demonstrating sustained drug release from Soluplus^®^ micelles. (**C**) Saturation solubility of FLQY2 as a function of drug-to-Soluplus^®^ ratio. (**D**) Plasma concentration–time curves after oral administration of FLQY2-SD (1:15 and 1:10), FLQY2 physical mixture (PM, 1:15), and cyclodextrin suspension in rats, illustrating the 12.3-fold BA enhancement achieved by the optimized SD formulation. (**E**) Tissue distribution of FLQY2 at various time points following oral administration. (**F**) Cumulative fecal and urinary excretion profiles of FLQY2. Reproduced from Wang et al. [73], Journal of Nanobiotechnology, 2022, CC BY 4.0 under the terms of the Creative Commons CC BY license.

Biochanin A, a natural isoflavone with anticancer properties but limited oral absorption, was similarly encapsulated in Pluronic F127/Plasdone S630 mixed micelles (Figure 5) [72]. The optimized formulation demonstrated a particle size of 25 nm, high encapsulation efficiency, and enhanced Caco-2 permeability with a reduced efflux ratio (0.96 vs. 1.63 for free drug), suggesting effective P-gp inhibition. Oral administration in rats yielded a 2.16-fold improvement in BA relative to biochanin A suspension.

Extending this concept to graft copolymers, Wang et al. demonstrated that a camptothecin analog (FLQY2) formulated as a Soluplus^®^ self-micelle solid dispersion achieved a 12.3-fold increase in oral BA compared with a cyclodextrin-based suspension in rats [73]. The Soluplus^®^-based system, prepared by solvent evaporation, spontaneously self-assembled into micelles in aqueous solution with dramatically improved solubility (332 µg/mL), demonstrating the potential of amphiphilic graft copolymers as alternatives to traditional PEO–*b*–PPO–*b*–PEO triblock poloxamers for oral micellar delivery.

Notably, Abdelbary and Makhlouf provided one of the few clinical demonstrations of poloxamer-based oral micelles, showing that dexibuprofen-loaded Pluronic F127 micelles formulated as lyophilized tablets achieved a relative oral BA of 160.15% compared with commercial tablets in a pharmacokinetic study involving six healthy human volunteers [74]. This result is significant because it demonstrates that polymeric micelle technology can enhance oral drug exposure not only in preclinical animal models but also in human subjects.

### 4.2. PEG–b–PLA Systems

PLA-based amphiphilic block copolymers, particularly PEG–*b*–PLA and PEG–*b*–PLLA systems, represent one of the most extensively studied platforms for oral micellar delivery. The hydrophobic polyester core enables efficient encapsulation of poorly soluble drugs, while the PEG corona provides steric stabilization and protection against aggregation and enzymatic degradation [75,76,77,78]. The semicrystalline nature of PLA cores and their relatively high glass-transition temperatures can contribute to dense core packing and slow chain-exchange kinetics, potentially conferring enhanced kinetic stability.

Quercetin-loaded mPEG-*b*-PLLA micelles prepared via thin-film hydration provide a representative example of their performance [78]. These micelles displayed particle sizes of approximately 89 nm and encapsulation efficiencies around 83%. Following oral administration in rats, the micellar formulation achieved an approximately 9-fold increase in oral BA compared with quercetin suspension. The improvement was associated with enhanced dissolution kinetics, protection from oxidative degradation in the intestinal environment, and prolonged retention in systemic circulation. By maintaining quercetin in a solubilized state during GI transit, the micelle effectively mitigated precipitation-driven absorption limitations.

In comparison, the enhancement was observed with US597, a ursolic acid derivative, encapsulated in PLGA–*b*–PEG–*b*–PLGA triblock copolymeric micelles [79]. In this case, oral administration resulted in approximately a 2.57-fold increase in AUC relative to free drug, accompanied by sustained in vitro release over 100 h. The semi-crystalline polyester core likely contributed to slower drug diffusion and improved kinetic stability under GI conditions. The substantial pharmacokinetic enhancement suggests that structural features such as core crystallinity and hydrophobic block length play critical roles in determining oral performance.

In a complementary study, Duan et al. developed curcumin-loaded mPEG–*b*–PLA/TPGS mixed micelles that exploited the dual functionality of TPGS as both a P-gp inhibitor and a co-emulsifier [80]. The mixed micellar system achieved a 9.27-fold increase in oral BA of curcumin in rats compared with curcumin suspension, which the authors attributed to enhanced intestinal permeability through TPGS-mediated efflux inhibition, possibly combined with improved micellar stability conferred by the PLA core, although in vivo integrity was not directly assessed. This result is notable because it demonstrates that polymer blending strategies can simultaneously address multiple absorption barriers. Collectively, PEG–*b*–PLA systems appear well aligned with stability-oriented design principles, as their cores (glassy amorphous (PDLLA) or semicrystalline (PLLA) cores) and tunable hydrophobic block lengths are expected to offer enhanced resistance to dilution and bile salt-induced destabilization, although this has not been systematically validated under biorelevant oral conditions [75,76].

### 4.3. PEG–b–PCL Systems

PCL-based block copolymers offer highly hydrophobic and relatively slow-degrading cores, which can support sustained drug release and stable encapsulation of lipophilic drugs [81]. The combination of PEG as a hydrophilic shell and PCL as a hydrophobic core produces micelles that are often structurally robust and capable of maintaining drug solubilization under challenging GI conditions.

Cyclosporine A-loaded PEG–*b*–PCL micelles represent a notable example [25]. In comparative oral pharmacokinetic studies, micellar formulations demonstrated approximately 2–3 fold increases in BA relative to conventional formulations. The enhancement was attributed to improved dissolution in bile salt-containing intestinal fluids, partial protection against chemical degradation, and possible contribution of lymphatic transport pathways. The hydrophobic PCL core provided strong drug–polymer interactions that stabilized cyclosporine A within the micelle, while the PEG corona minimized aggregation and facilitated mucus diffusion. Similarly, PEG–*b*–PDLLA micelles have been explored for both doxorubicin (via acid-cleavable conjugation) [82] and paclitaxel [83] via intravenous administration, showing improved systemic exposure and reduced toxicity compared with Cremophor-based formulations.

Among the most striking pharmacokinetic enhancements reported for PEG–*b*–PCL systems, Manjili et al. demonstrated that curcumin-loaded mPEG–*b*–PCL micelles (81 nm, 89.3% EE) achieved a remarkable 52.8-fold increase in oral AUC compared with curcumin suspension in rats [84]. The authors attributed this exceptional enhancement to the combination of improved aqueous solubility, sustained drug release from the hydrophobic PCL core, and enhanced intestinal permeability potentially conferred by the micellar nanostructure, although the direct evidence of intact micelle was not provided.

Additionally, Wang et al. developed an innovative PEG–*b*–PCL micelle-hydrogel composite system for oral docetaxel delivery that achieved 75.6% absolute oral BA in rats, representing a 10-fold improvement over conventional formulations [85]. The hydrogel matrix provided gastric protection while enabling sustained micelle release in the intestinal environment. Because PCL exhibits greater hydrophobicity and slower degradation than PLA, PEG–*b*–PCL systems may offer improved kinetic stability during intestinal transit. However, the slower degradation rate necessitates careful evaluation of long-term safety and clearance, particularly for repeated oral dosing regimens [81].

### 4.4. PEG–b–PLGA Systems

PLGA-based amphiphilic block copolymers, particularly PEG–*b*–PLGA diblock and PLGA–*b*–PEG–*b*–PLGA triblock architectures, have attracted considerable attention for oral micellar delivery owing to their tunable degradation rates, which are determined by the lactide-to-glycolide (LA/GA) ratio, and their established biocompatibility and regulatory precedent [86].

Oral pharmacokinetic studies have demonstrated substantial BA enhancements with PEG–*b*–PLGA micellar formulations. Andrographolide-loaded PLGA–*b*–PEG–*b*–PLGA triblock micelles (124 nm, 8.4% drug loading) achieved a 2.7-fold increase in plasma AUC and 2.5-fold longer mean residence time compared with free drug suspension [87]. Similarly, 2-methoxyestradiol encapsulated in PEG–*b*–PLGA micelles (58 nm) exhibited an oral absolute BA of approximately 50%, which was further elevated to over 120% when the micelles were incorporated into pH-responsive microspheres [88]. These results suggest that the combination of micellar solubilization with gastro-protective strategies can synergistically enhance oral absorption of poorly bioavailable compounds.

Additional in vivo evidence supports the versatility of PLGA–*b*–PEG–*b*–PLGA triblock architectures. Song et al. prepared curcumin-loaded PLGA–*b*–PEG–*b*–PLGA micelles (26 nm) by a thin-film hydration method and evaluated their pharmacokinetics in rats after oral administration. The micellar formulation significantly prolonged the terminal half-life (4.54-fold increase) and extended the mean residence time by 2.67-fold compared with a curcumin suspension, indicating sustained absorption and delayed elimination attributable to the triblock micellar structure [89]. These findings complement the andrographolide [87] and 2-methoxyestradiol data cited above and reinforce PEG–*b*–PLGA as a robust platform for improving the oral pharmacokinetics of biopharmaceutical classification system (BCS) class II/IV compounds.

A key advantage of PEG–*b*–PLGA systems relative to PEG–*b*–PLA and PEG–*b*–PCL lies in their capacity to accommodate both hydrophilic and hydrophobic drug molecules within the same polymer platform. PEG–*b*–PLGA micelles have demonstrated encapsulation efficiencies ranging from 56% to 79% for diverse small molecules including curcumin, paclitaxel, and andrographolide, with drug-loading capacities typically between 8% and 29% depending on the formulation method [79,88]. The faster hydrolytic degradation of the glycolide component relative to lactide provides an additional mechanism for controlled drug release in the GI environment, making PEG–*b*–PLGA micelles a versatile platform for oral delivery of poorly water-soluble drugs.

Emerging evidence also supports the integration of active targeting and stimuli-responsive strategies within PEG–*b*–PLGA-based micellar systems. Beyond PLGA-based approaches, Sun et al. developed OCTN2-targeted, ROS-responsive hybrid polymeric micelles based on PEG–PCL architecture by blending L-carnitine-grafted, thioketal-linked LC–*b*–PEG_55k_-TK-PCL_7k_ with mPEG_5k_-PCL_7k_ for oral docetaxel delivery [90]. The optimized hybrid formulation demonstrated 3.37-fold higher transcytosis than non-targeted controls and achieved a 10-fold increase in oral BA in mice, while orally administered micelles (40 mg/kg) produced comparable antitumor efficacy with reduced systemic toxicity relative to intravenous docetaxel injection (10 mg/kg). This study suggests that next-generation oral polymeric micelles incorporating both transporter-mediated uptake and microenvironment-responsive drug release may substantially improve the therapeutic index of cytotoxic agents, although confirmation in larger animal models is warranted.

### 4.5. Elimination of Polymers

Comprehensive understanding of the metabolism and excretion of the polymer is critical for establishing the safety of oral polymeric micelle systems. Biodegradable polyesters undergo hydrolytic degradation into their respective monomers: PLA into lactic acid, PLGA into lactic and glycolic acids [91] and PCL into 6-hydroxyhexanoic acid [92]. Lactic acid enters the tricarboxylic acid (TCA) cycle and is ultimately eliminated as CO_2_ and H_2_O, while glycolic acid is metabolized similarly or excreted renally unchanged. 6-Hydroxyhexanoic acid is primarily metabolized through normal catabolic pathways. The degradation kinetics of these polyesters are well-characterized and form the basis for USA Food and Drug Administration (FDA) approval of numerous PLA- and PLGA-based products [91]. Low molecular weight PEG (<20 kDa) is excreted renally; PEG used in oral micelle formulations is typically 2–5 kDa, well below the renal clearance threshold. PEG has Generally Recognized As Safe (GRAS) status and extensive safety data supporting its use in pharmaceutical applications [93]. Poloxamers are essentially non-biodegradable; following intravenous administration, they are cleared renally, but after oral administration, the majority of unabsorbed poloxamer transits the GI tract and is excreted in feces [94]. Indeed, for all polymer classes discussed here, oral administration results in limited systemic absorption, with most polymer remaining in the GI lumen and undergoing fecal excretion, thereby minimizing concerns regarding systemic toxicity compared to intravenous administration. Comprehensive understanding of the metabolism and excretion of the polymer is critical for establishing the safety of oral polymeric micelle systems.

**Table 1:** Comparative overview of amphiphilic block copolymer systems employed for oral polymeric micelle formulations.

Polymer/Drug	Formulation	Stability Evidence	BA Enhancement	Ref
Pluronic: Non-biodegradable; fecal excretion (oral), renal (if absorbed)|Key: P-gp inhibition; simple formulation|Limitation: high CMC → dilution-sensitive				
Pluronic F127/Genistein	28 nm; EE ~97%; Solid dispersion	Size/EE only; P-gp inhibition inferred from enhanced absorption	Enhanced ↑ (not quantified; rat)	[70]
Pluronic P123/F127/Lacidipine	21 nm; EE ~100%; CCD-optimized	Size/EE/dissolution only; >96% dissolution in 10 min; P-gp inhibition	6.85-fold (rabbit)	[65]
Pluronic F127/Plasdone S630/Biochanin A	25 nm; EE high; Thin-film hydration	Caco-2 efflux ratio 0.96 vs. 1.63 (free drug); P-gp inhibition confirmed	2.16-fold (rat)	[72]
Pluronic F127/Dexibuprofen	Lyophilized tablet	Size/dissolution only; reconstitution from solid form	1.60-fold (human, n = 6)	[74]
PEG–*b*–PLA|Hydrolysis → lactic acid (TCA cycle → CO_2_/H_2_O); PEG: renal excretion|Key: dense semicrystalline core; slow chain exchange; lower hydrophobicity than PCL				
mPEG–*b*–PLLA/Quercetin	88.5 nm; EE ~83%; Thin-film hydration	Size/EE only; protection from oxidative degradation inferred	~9× (rat)	[78]
mPEG–*b*–PLA/TPGS/Curcumin	—	Size/EE only; TPGS-mediated P-gp inhibition confirmed separately	9.27× (rat)	[80]
PEG–b–PCL: Slow hydrolysis → 6-hydroxycaproic acid; PEG: renal excretion|Key: highly hydrophobic core; high kinetic stability; slow degradation				
PEG–*b*–PCL/Cyclosporine A	2-3NT (28 nm); 5-7E (112 nm)	FRET: intact micelle detected in intestinal tissue; bile salt “solubilization effect” & mucin “hydration effect” characterized; PCL block length modulates integrity via competing mechanisms	1.5-fold (rat)	[25]
PEG–*b*–PCL/Curcumin	81 nm; EE 89.3%	Size/EE only; high EE from strong drug–PCL hydrophobic interaction	52.8-fold (rat)	[84]
PEG–*b*–PCL (OCTN2-targeted, ROS-responsive)/Docetaxel	20 nm; EE 97.02%; DL 7.76%	ROS-responsive release; antitumor efficacy confirmed	10× (mouse)	[90]
PEG–b–PLGA: Hydrolysis → lactic + glycolic acid; PEG: renal excretion|Key: tunable degradation; broad drug compatibility; faster degradation may reduce kinetic stability vs. PCL				
PLGA–*b*–PEG–b–PLGA US597 (ursolic acid deriv.)	DL 25.9–28.5%	Sustained release > 100 h; semicrystalline core likely contributed to kinetic stability	~2.57-fold (rat)	[79]
PLGA–b–PEG–b–PLGA/Andrographolide	124 nm; DL 8.4%	Size/DL only; MRT 2.5× prolonged suggests structural persistence	2.7× (rat)	[87]
PEG–b–PLGA (+pH-resp. MS)/2-Methoxyestradiol	58 nm; DL 7.9%	pH-responsive microsphere: gastric protection; micellar + gastro-protective synergy	2.43-fold (rat)	[88]
PLGA–b–PEG–b–PLGA/Curcumin	26 nm Thin-film hydration	Size only; prolonged t½ and MRT suggest sustained absorption	1.31-fold (rat)	[89]

## 5. Comparative Analysis: Polymeric Micelles, SMEDDS, and ASDs

To contextualize the advantages and limitations of polymeric micelles for oral drug delivery, a direct comparison with the two most commercially successful alternative platforms such as SMEDDSs and ASDs is warranted. Table 2 summarizes the key differentiating attributes across critical formulation parameters [24,29,62,95,96].

As illustrated in Table 2, polymeric micelles occupy a unique position among oral solubility-enhancing platforms in that they are the only system that combines nanoscale dimensions with the potential for surface engineering and nanocarrier-mediated transport. However, this advantage is realized only when micelle integrity is maintained through the GI tract—a condition that, as discussed in Section 3, requires deliberate engineering of core stability.

SMEDDSs, which underpin commercially successful products such as Neoral^®^ (cyclosporine A), offer robust solubilization capacity for lipophilic drugs without the structural integrity concerns that limit polymeric micelles. Upon contact with aqueous GI fluids, SMEDDSs spontaneously form fine oil-in-water emulsions that present the drug in a pre-solubilized state, circumventing dissolution-limited absorption [29]. Their manufacturing is straightforward, requiring only the mixing of lipid, surfactant, and co-surfactant components, and scale-up is well established. However, SMEDDS formulations are inherently limited to lipophilic compounds, rely on high surfactant loads that may cause GI irritation, and offer no mechanism for surface functionalization or targeted mucosal interaction [95]. Recent efforts to overcome these limitations include solidified SEDDS formulated with mesoporous carriers for improved stability and handling [97], as well as integrative strategies that combine SEDDS with nanoparticulate technologies to achieve controlled release and enhanced targeting capabilities [98]. Furthermore, SMEDDS performance is highly sensitive to the prandial state, with bile salt and lipid composition significantly affecting emulsification efficiency and drug solubilization capacity [24,29].

ASDs, exemplified by marketed products such as Kalydeco^®^ (ivacaftor) and Sporanox^®^ (itraconazole), achieve BA enhancement through a fundamentally different mechanism. The drug is molecularly dispersed within a hydrophilic polymer matrix, generating transient supersaturation upon dissolution that drives enhanced passive absorption [95,96]. ASDs can be manufactured at industrial scale via hot-melt extrusion or spray drying with established quality control frameworks [95]. For example, a spray-dried solid dispersion of chrysin with sodium dodecyl sulfate and polyvinylpyrrolidone significantly enhanced oral BA by simultaneously improving solubility and inhibiting intestinal first-pass metabolism [99]. Their principal limitation is physical instability in that the thermodynamically unfavorable amorphous state tends toward recrystallization during storage, particularly under elevated temperature and humidity conditions [95]. Notably, neither SMEDDS nor ASDs offer nanocarrier-mediated transport advantages such as endocytic uptake, M-cell transcytosis, or lymphatic absorption, which remain the principal mechanistic differentiators of structurally intact polymeric micelles [62].

Despite these theoretical advantages, head-to-head comparisons between polymeric micelles and competing platforms remain scarce. Only one direct comparison between a polymeric micelle formulation and an ASD for the same drug (paclitaxel) has been reported [96]. In that study, the optimized solid dispersion formulation achieved higher oral BA than the polymeric micelle (667% vs. 365% relative to unformulated paclitaxel), although both formulations demonstrated comparable in vitro anticancer effects and no analogous comparison with SMEDDS was identified in the literature surveyed [96]. This paucity of comparative data makes it difficult to determine whether the added complexity and cost of micellar formulations are justified for a given drug candidate. From a practical formulation selection standpoint, polymeric micelles may offer unique advantages for specific drug candidates: compounds requiring efflux transporter modulation (e.g., Pluronic-mediated P-gp inhibition, which has been molecularly demonstrated in Caco-2 permeability studies [72]), drugs requiring protection from GI degradation through core encapsulation, and drugs where sustained supersaturation maintenance is critical for absorption. Conversely, alternative platforms may be more practical in other scenarios: SMEDDS for drugs where solubilization alone suffices for therapeutic exposure, given their established manufacturing and regulatory pathways; ASDs for thermally stable compounds amenable to hot-melt extrusion with proven industrial scalability; and development programs prioritizing time-to-market with well-characterized quality control frameworks [29,95,96]. However, these selection considerations remain largely based on mechanistic reasoning rather than direct comparative evidence, underscoring the need for systematic head-to-head studies.

## 6. Clinical Development and Translational Perspectives

Despite extensive preclinical research demonstrating the potential of polymeric micelles for oral drug delivery, no oral polymeric micelle product has yet achieved regulatory approval [100,101]. By contrast, the clinical development of intravenous polymeric micelle formulations has progressed considerably. Genexol-PM^®^, a PEG-*b*-PDLLA micelle encapsulating paclitaxel developed by Samyang Biopharmaceuticals, received marketing approval in South Korea in 2006 for breast and non-small cell lung cancer, becoming the first commercially available polymeric micelle product. This Cremophor EL-free formulation demonstrated improved safety profiles with reduced hypersensitivity reactions while maintaining antitumor efficacy comparable to conventional paclitaxel; however, it has not received FDA approval owing to concerns regarding the absence of a clear bioequivalence advantage in pivotal trials [62,101]. NK105, a PEG–*b*–poly(aspartate) micellar paclitaxel formulation developed by NanoCarrier Co., reached Phase III clinical trials in Japan for metastatic breast cancer but failed to demonstrate superiority over conventional paclitaxel in the primary endpoint of progression-free survival [62,101]. NC-6004 (Nanoplatin^®^), a PEG–*b*–poly(glutamic acid) micelle encapsulating cisplatin, has completed Phase III trials in combination with gemcitabine for pancreatic cancer, with results showing reduced nephrotoxicity though modest efficacy improvements [101]. As of 2020, more than 15 polymeric micelle formulations had entered clinical trials, predominantly for parenteral oncology applications, with none specifically designed for oral administration reaching advanced clinical stages [62].

This stark disparity between intravenous and oral clinical progress reflects the fundamentally different biophysical environments encountered by each route. As summarized in Table 3, the oral route introduces a constellation of constraints including massive and variable dilution by GI secretions, exposure to 2–15 mM competing bile salt surfactants, a viscoelastic mucus barrier, dynamic pH gradients from 1.2 to 7.4, and a narrow 3–5 h small intestinal transit window that collectively challenge micelle integrity in ways absent from the bloodstream [24,62].

The success of intravenous polymeric micelles in reaching clinical approval underscores that the micellar technology platform itself is viable; however, the oral route demands not merely incremental improvements in formulation parameters, but micelles specifically engineered for the GI environment from the outset. This stands in contrast to competing formulation platforms that have already achieved regulatory success: ASDs have enabled multiple FDA-approved products including Kalydeco^®^ (ivacaftor) and Orkambi^®^ (lumacaftor/ivacaftor), while SMEDDS underpin the commercially successful Neoral^®^ (cyclosporine) formulation [29,95,96].

### Bridging the Oral Translation Gap

Closing the translational gap between preclinical promise and clinical reality for oral polymeric micelles requires addressing several interconnected challenges. First, the lack of standardized in vitro models that accurately replicate the complexity of the GI environment including dynamic pH gradients, bile salt fluctuations, enzymatic activity, and mucus turnover limits the predictive value of preclinical screening [24,25]. The development and adoption of biorelevant dissolution media (FaSSIF/FeSSIF), multi-compartment GI simulators, and organ-on-chip platforms could improve the correlation between in vitro performance and in vivo outcomes. In parallel, advances in pharmacokinetic modeling, including physiologically based pharmacokinetic (PBPK) and population PK approaches, offer increasingly powerful tools for predicting in vivo disposition from in vitro formulation data, thereby accelerating rational formulation development [102]. Second, regulatory frameworks for oral nanomedicines remain evolving; the FDA’s draft guidance on liposomal drug products (2018) provides some precedent, but specific guidance for polymeric micelle oral formulations has not been established [62]. Key regulatory considerations include defining critical quality attributes (CQAs) linked to micelle integrity, demonstrating stability in biorelevant GI media, and establishing appropriate dissolution specifications that correlate with in vivo pharmacokinetic performance.

Third, the economic viability of oral polymeric micelles must be demonstrated through pharmacoeconomic analyses comparing the cost-effectiveness of micellar formulations against established SMEDDS and ASD technologies that have proven manufacturing scalability [29,95,96,103,104]. Reproducible large-scale manufacturing and compliance with current Good Manufacturing Practice (cGMP) requirements represent additional prerequisites for clinical translation. Al-Lawati and Binkhathlan recently highlighted that the translation of oral polymeric micelles to marketed products remains contingent on demonstrating clear therapeutic advantages over simpler formulation approaches in well-designed clinical trials [24]. Bridging this gap will ultimately require standardized testing methodologies that explicitly assess micelle integrity under physiologically relevant conditions and correlate these measurements with in vivo pharmacokinetic outcomes.

**Figure 6:**
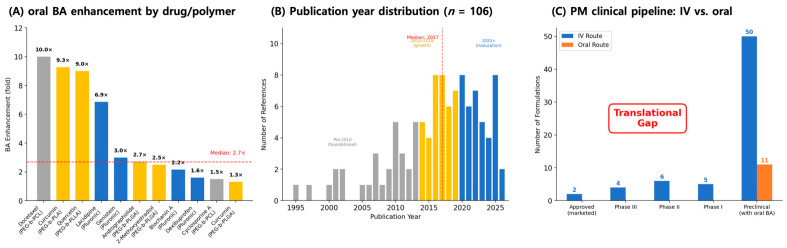
Quantitative analysis of oral polymeric micelle literature. (**A**) Oral bioavailability enhancement (fold-increase vs. control) across drug/polymer combinations. Dashed red line indicates median (2.7×). (**B**) Publication year distribution of the 106 references analyzed in this review (median: 2017). (**C**) Comparison of IV and oral polymeric micelle clinical development pipelines, illustrating the translational gap in oral delivery.

To provide a quantitative foundation for this review, we analyzed the preclinical pharmacokinetic data reported across the oral polymeric micelle studies discussed herein (Figure 6). Across 11 formulations spanning four polymer classes in Table 1, the median oral BA enhancement was 2.7-fold relative to either unformulated controls or free drug. Despite this growing preclinical evidence base, with 64% of references published after 2015, a stark translational gap persists: more than 15 intravenous polymeric micelle formulations have reached clinical trials, yet no oral polymeric micelle product has entered clinical development as of 2025 [62,101]. Furthermore, 92% of the quantitative BA studies relied on rodent models, only one direct head-to-head comparison with ASD was identified [96], no comparisons with SMEDDS were found, and mechanistic deconvolution distinguishing nanocarrier-mediated transport from solubilization-driven absorption was attempted in only a minority of studies. These quantitative observations underscore the need for both critical reassessment of the current literature and a clearly defined roadmap for clinically meaningful oral polymeric micelle development.

## 7. Critical Perspectives on Oral Polymeric Micelle Research

While previous sections have presented the current state of oral polymeric micelle research, several fundamental assumptions underlying this field warrant critical examination. The following perspectives highlight key conceptual limitations that, if unaddressed, may continue to impede meaningful clinical progress.

### 7.1. Nanocarriers Versus Solubilizers: Reassessing the Functional Role of Oral Micelles

A critical limitation in the current literature is the implicit assumption that polymeric micelles function as intact nanocarriers following oral administration. In reality, the harsh GI environment, characterized by bile salt competition, extreme dilution, pH fluctuations, and enzymatic activity, presents formidable challenges to micellar structural integrity [9,105]. In many cases, micelle disassembly likely occurs prior to epithelial interaction, suggesting that the observed BA enhancement may arise primarily from transient solubilization and supersaturation rather than nanoparticle-mediated transport [21,60]. Enhanced oral BA is frequently attributed to nanoparticle-mediated mechanisms including transcytosis and lymphatic uptake; however, such interpretations are often not rigorously supported by mechanistic evidence distinguishing intact micellar transport from molecular-level absorption of solubilized drug [105]. Without direct evidence that micelles remain structurally intact at the absorptive epithelium, claims of nanocarrier-mediated oral delivery should be interpreted with caution.

### 7.2. Limitations of CMC as a Stability Descriptor

The widespread reliance on CMC as a predictor of in vivo micelle stability may be overly simplistic [9,102]. GI conditions are inherently non-equilibrium systems, where continuous dilution, bile salt influx, and food-related compositional changes occur simultaneously [27]. Even systems with exceptionally low CMC values may undergo rapid structural rearrangement or complete disassembly due to bile salt penetration into the micellar core and competitive displacement of encapsulated drug [63]. These observations highlight the importance of kinetic stability rather than thermodynamic parameters alone [9,27,106]. Moreover, the practice of ranking polymer classes by their “typical” CMC ranges may be less reliable because CMC is primarily governed by the hydrophobic block length and molecular weight rather than by the chemical identity of the core-forming polymer. No systematic study has compared CMC values across different polymer types under matched block-length and molecular-weight conditions; therefore, cross-platform CMC rankings reported in the literature should not be taken as intrinsic properties of a given polymer class. Future studies should therefore incorporate kinetic stability assessments as essential complements to CMC determination.

### 7.3. Interpretive Challenges in FRET-Based Integrity Studies

FRET-based approaches have become the de facto standard for probing polymeric micelle integrity in biological environments [17,20]. However, these methods are not without significant limitations. FRET signal loss may reflect hydrophobic probe redistribution to lipoproteins, cell membranes, or other biological sinks rather than complete micelle disassembly [60,61]. Chen et al. demonstrated that lipophilic FRET donors can transfer to cell membranes faster than the polymer carrier itself is internalized, creating an artifactual impression of rapid micelle disintegration [61]. Furthermore, Zhang et al. showed that conventional FRET pairs suffer from self-quenching artifacts that lead to inaccurate stability measurements [20]. Guo et al. further demonstrated that FRET-derived integrity data are highly sensitive to the physicochemical properties of the probe pair, potentially overestimating or underestimating true structural stability [17]. More recently, Zhang et al. employed direct nanocarrier isolation via density gradient ultracentrifugation coupled with aggregation-caused quenching probes, providing a more reliable assessment of in vivo micellar integrity [107]. These findings collectively underscore the need for multi-modal characterization approaches that go beyond FRET alone.

### 7.4. Questioning the Necessity and Added Value of Micellar Systems

Given that simpler formulation platforms such as SMEDDSs and ASDs can achieve comparable or superior solubilization and BA enhancement for poorly soluble drugs [29,95,96], the added complexity of polymeric micelle formulations must be justified by clear mechanistic advantages. As discussed in Section 5 of this review, head-to-head comparisons between polymeric micelles and these competing platforms remain scarce, and the available evidence does not consistently demonstrate superiority of micellar systems for oral delivery [62]. The multi-step preparation processes, stringent quality control requirements, and stability challenges associated with polymeric micelles impose additional manufacturing burdens that must be weighed against any incremental therapeutic benefit. For oral polymeric micelles to be commercially viable, future research must demonstrate unique advantages—such as targeted mucosal interaction, controlled supersaturation kinetics, or stimuli-responsive release—that cannot be readily achieved by simpler formulation approaches.

### 7.5. Translational Failure and Conceptual Mismatch

The absence of clinically approved oral polymeric micelle products, despite decades of preclinical research, may reflect not merely technical limitations but a fundamental conceptual mismatch between the assumptions of micellar drug delivery and the realities of the GI environment [108,109,110]. The nanomedicine field broadly has faced a translational crisis: of the thousands of nanoformulations reported in the literature, only a handful have reached clinical use, and most of these are parenteral products [108]. For oral delivery specifically, the dynamic and destructive nature of the GI tract imposes constraints that are qualitatively different from the circulatory system, where most successful nanomedicines operate [108,109]. Bridging this gap will require the oral polymeric micelle field to move beyond demonstrating in vitro stability and preclinical BA enhancement toward establishing genuine mechanistic understanding of micellar fate in the GI tract, supported by validated analytical tools that can track intact nanostructures in physiologically relevant conditions [27,107]. In parallel, emerging polymer platforms such as poly(2-oxazoline) (POx)-based micelles, which offer exceptionally high drug-loading capacity (~50 wt%) and stealth properties comparable to PEG without anti-PEG immunogenicity concerns, warrant further investigation for oral applications [111,112,113]; however, the complete absence of published oral pharmacokinetic data for POx systems underscores that rigorous in vivo validation remains a prerequisite before these materials can be considered viable oral delivery platforms.

BCS Class II (low solubility, high permeability) and Class IV (low solubility, low permeability) compounds with acceptable intestinal stability are ideal can be candidates for oral polymeric micelle delivery [14,62]. Examples include curcumin, paclitaxel, cyclosporine A, and quercetin featured in our review or therapeutic compounds for chronic conditions requiring repeated dosing (e.g., anti-inflammatory, metabolic, or chemopreventive agents). In contrast, compounds requiring precise dose control (e.g., cytotoxic agents with narrow therapeutic windows), drugs or with severe GI degradation, or drugs targeting systemic tumors where direct within bloodstream access are better served by IV micellar formulations, as exemplified by is critical. A product such as Genexol-PM^®^ (paclitaxel) exemplify this category [62,83]. Namely, The choice between oral and IV routes depends on the drug’s therapeutic index, GI stability, target tissue, required onset of action, and patient compliance considerations [15,77]. Oral micelles are most advantageous for chronic conditions requiring repeated dosing (e.g., anti-inflammatory, metabolic, or chemopreventive agents) [24,77].

## 8. Conclusions

This review has argued that the central challenge facing oral polymeric micelles is not formulation optimization per se, but a more fundamental question of whether a given micellar system functions as a true nanocarrier or merely as a transient solubilizer in the hostile GI environment. The evidence from FRET-based integrity studies, bile salt competition assays, and comparative pharmacokinetic analyses consistently demonstrates that only micelles engineered with high core stability, characterized by sufficient hydrophobic block length to achieve low CMC values, crystalline or high-Tg cores, and optimized PEG corona density, can maintain structural integrity through the GI tract and potentially deliver drugs via nanocarrier-mediated transport mechanisms, although definitive clinical proof of intact micelle transport in humans remains to be established.

From a translational standpoint, the pharmaceutical community should critically evaluate whether the added complexity and cost of micellar formulations are justified over established alternatives such as SMEDDS and ASDs for systems that primarily function as solubilizers. For micelle systems that demonstrate structural persistence, the path forward requires standardized biorelevant testing protocols that assess micelle integrity under physiologically representative conditions, coupled with head-to-head clinical comparisons against conventional oral formulations. The future of oral polymeric micelles will depend not on incremental formulation refinement but on fundamentally rethinking micelle stability in the context of GI thermodynamics, biophysical competition, and translational feasibility.

## Figures and Tables

**Table 2:** Comparative overview of polymeric micelles, SMEDDS, and ASDs for oral drug delivery of poorly water-soluble small molecules.

Parameter	Polymeric Micelles	SMEDDS	ASD
Mechanism	Self-assembly of amphiphilic block copolymers into core–shell nanostructures	Spontaneous microemulsification upon GI dilution	Molecular dispersion of drug in polymer matrix; supersaturation upon dissolution
Typical size	10–50 nm	100–300 nm (after emulsification)	N/A (molecular dispersion)
Solubilization	Moderate; dependent on core–drug compatibility (Flory–Huggins χ)	High; broad applicability for lipophilic drugs	High; achieves supersaturation
GI stability	Critical structural vulnerability: bile salt destabilization and dilution-induced disassembly	Dependent on GI fluid volume and composition	Risk of recrystallization; moisture-sensitive
Surface engineering	Highly versatile: PEGylation, targeting ligands, stimuli-responsive	Limited	Not applicable
Nanocarrier function	Only if integrity maintained; may enables transcytosis, lymphatic uptake, and efflux inhibition	No; solubilization only	No; supersaturation only
Manufacturing	Moderate complexity; dialysis, thin-film, or direct dissolution	Simple; mixing of liquid components	Specialized: hot-melt extrusion, spray drying
Scale-up readiness	Challenging; batch-to-batch variability	Established; multiple marketed products (e.g., Neoral^®^)	Established; multiple marketed products (e.g., Sporanox^®^)
Key limitation	Low drug loading capacity; dynamic equilibrium threatens structural identity; high polymer cost	GI irritation from high surfactant load; high inter-subject variability from food effects	Physical instability; recrystallization during storage
Clinical status (oral)	No approved oral products to date despite decades of preclinical research	Multiple approved products	Multiple approved products

**Table 3:** Comparative biophysical constraints: intravenous vs. oral polymeric micelle delivery.

Feature	IV Micelle	Oral Micelle
Dilution	Controlled (blood volume)	Massive and variable (GI secretions)
Bile salts	None	2–15 mM competing surfactants
Mucus barrier	Absent	Viscoelastic barrier requiring penetration
pH range	7.4 (constant)	1.2–7.4 (dynamic gradient)
Transit time constraint	None (circulation half-life)	3–5 h (small intestine window)
Protein corona	Plasma proteins (well-characterized)	Gut corona (poorly understood)
Regulatory precedent	Genexol-PM^®^ approved [54,85]	No approved products

## Data Availability

There are no data to support the findings of this review.

## Abbreviations

ASD	Amorphous Solid Dispersion
AUC	Area Under the Curve
BA	Bioavailability
BCS	Biopharmaceutical Classification System
cGMP	Current Good Manufacturing Practice
CMC	Critical Micelle Concentration
CQAs	Critical Quality Attributes
DL	Drug Loading
EE	Encapsulation Efficiency
FaSSIF	Fasted State Simulated Intestinal Fluid
FDA	Food and Drug Administration
FeSSIF	Fed State Simulated Intestinal Fluid
FRET	Förster Resonance Energy Transfer
GI	Gastrointestinal
GRAS	Generally Recognized As Safe
LA/GA	Lactide-to-Glycolide (ratio)
MW	Molecular Weight
OCTN2	Organic Cation/Carnitine Transporter 2
PCL	Poly(ε-caprolactone)
PDLLA	Poly(D,L-lactic acid)
PEG	Poly(ethylene glycol)
PEO	Poly(ethylene oxide)
P-gp	P-glycoprotein
PLA	Poly(lactic acid)
PLGA	Poly(lactic-co-glycolic acid)
PLLA	Poly(L-lactic acid)
PPO	Poly(propylene oxide)
SAXS	Small-Angle X-ray Scattering
SMEDDS	Self-Microemulsifying Drug Delivery Systems
TEM	Transmission Electron Microscopy
Tg	Glass Transition Temperature
TPGS	D-α-Tocopheryl Polyethylene Glycol 1000 Succinate
